# The boundary of life and death: changes in mitochondrial and cytosolic proteomes associated with programmed cell death of *Arabidopsis thaliana* suspension culture cells

**DOI:** 10.3389/fpls.2023.1194866

**Published:** 2023-08-01

**Authors:** Johanna Schwarze, James C. Carolan, Gavin S. Stewart, Paul F. McCabe, Joanna Kacprzyk

**Affiliations:** ^1^ School of Biology and Environmental Science, University College Dublin, Dublin, Ireland; ^2^ Department of Biology, Maynooth University, Maynooth, Ireland

**Keywords:** plant programmed cell death, mitochondria, proteomics, heat stress, cytosol

## Abstract

**Introduction:**

Despite the critical role of programmed cell death (PCD) in plant development and defense responses, its regulation is not fully understood. It has been proposed that mitochondria may be important in the control of the early stages of plant PCD, but the details of this regulation are currently unknown.

**Methods:**

We used *Arabidopsis thaliana* cell suspension culture, a model system that enables induction and precise monitoring of PCD rates, as well as chemical manipulation of this process to generate a quantitative profile of the alterations in mitochondrial and cytosolic proteomes associated with early stages of plant PCD induced by heat stress. The cells were subjected to PCD-inducing heat levels (10 min, 54°C), with/without the calcium channel inhibitor and PCD blocker LaCl_3_. The stress treatment was followed by separation of cytosolic and mitochondrial fractions and mass spectrometry-based proteome analysis.

**Results:**

Heat stress induced rapid and extensive changes in protein abundance in both fractions, with release of mitochondrial proteins into the cytosol upon PCD induction. In our system, LaCl_3_ appeared to act downstream of cell death initiation signal, as it did not affect the release of mitochondrial proteins, but instead partially inhibited changes occurring in the cytosolic fraction, including upregulation of proteins with hydrolytic activity.

**Discussion:**

We characterized changes in protein abundance and localization associated with the early stages of heat stress-induced PCD. Collectively, the generated data provide new insights into the regulation of cell death and survival decisions in plant cells.

## 1 Introduction

Programmed cell death (PCD) is a genetically regulated pathway for selective elimination of redundant, damaged, or infected cells (Danon et al., 2000). In plants, PCD plays a critical role in development (Daneva et al., 2016) and responses to abiotic and biotic stimuli (Yanık et al., 2020). Carefully regulated PCD events are, therefore, essential during the normal plant life cycle and can promote survival under stressful environmental conditions. The past two decades have witnessed significant advances in the field of plant PCD research, for example, in terms of elucidating the transcriptional regulation of this important process (Cubría-Radío and Nowack, 2019; Burke et al., 2020) or the identification of plant-specific proteases that execute the cellular demise (Balakireva and Zamyatnin, 2019). However, our understanding of the sequence of events leading to activation of plant PCD is still fragmented, especially in comparison to well-characterized animal cell death pathways.

The mitochondrion is a central signaling nexus between different cell death modalities in animals (Bock and Tait, 2020). Apoptosis, the most studied form of regulated animal cell death, can occur through two interconnected pathways depending on the cell death initiation signal: mitochondrial (intrinsic) pathway and death receptor–mediated (extrinsic) pathway (Hengartner, 2000). During apoptosis, interactions between pro- and anti-apoptotic members of the B cell lymphoma 2 (BCL-2) family result in BAX-mediated and BAK-mediated (pro-apoptotic BCL-2 proteins) permeabilization of outer mitochondrial membrane (OMM) and, consequently, the release of soluble proteins from the intermembrane mitochondrial space (IMS) into the cytosol that initiates a signaling cascade leading to cell death (Green and Llambi, 2015). Upon its release to the cytosol, cytochrome c (cyt *c*) triggers a cascade of catalytic activation of caspases that drive proteolytic degradation of the cell (Cain et al., 2002). Other released IMS proteins, such as SMAC (second mitochondria-derived activator of caspase) and Htra2/Omi (high temperature requirement 2), further facilitate apoptosis by blocking the inhibitor of apoptosis proteins such as the caspase inhibitor X-linked inhibitor of apoptosis protein (XIAP) (Du et al., 2000; van Loo et al., 2002; Muñoz-Pinedo et al., 2006). In addition, apoptosis-inducing factor (AIF), yet another protein released from the IMS upon apoptotic insult, can activate caspase-independent cell death after translocation to the nucleus where it triggers chromatin condensation and large-scale DNA degradation (Sevrioukova, 2011). More recently, involvement of the mitochondrion in other forms of regulated cell death, including necroptosis, ferroptosis, and pyroptosis, has been also reported (Bock and Tait, 2020), further underscoring the central role of this organelle in orchestrating the cell death programs operating in the animal kingdom.

Although the homologs of the core animal apoptotic machinery including the BCL-2 family and caspases have not yet been found in plant genomes, numerous studies strongly suggest that the mitochondrion also plays a key PCD role in plants. Early in the PCD process, bursts of mitochondrial reactive oxygen species (ROS) are commonly observed in plant cells (Gao et al., 2008; Bi et al., 2009), and, similarly to animal cells, their mitochondria undergo a permeability transition, swelling, and loss of transmembrane potential. Moreover, the chemicals that inhibit these mitochondrial changes can prevent the cell death process in plants, e.g., in sycamore (Contran et al., 2007), zinnia (Yu et al., 2002), *Arabidopsis* (Gao et al., 2008; Scott and Logan, 2008), lace plant (Lord et al., 2013) and *Micrasterias* alga (Darehshouri et al., 2008). The rapid release of cyt *c* into the cytosol, a key event in animal apoptosis, has been shown to occur in a variety of plant species, including cucumber (Balk et al., 1999), sunflower (Balk and Leaver, 2001), wheat (Virolainen et al., 2002), *Arabidopsis* (Yao et al., 2004), and cannabis (Morimoto et al., 2007). However, the role of cyt *c* in the plant PCD process in plants is debatable, as, in contrast to animal studies (Slee et al., 1999), cyt *c* alone was not sufficient to trigger DNA laddering, a hallmark feature of PCD, in an *Arabidopsis* cell-free system (Balk et al., 2003). Nevertheless, in the same study, the broken mitochondria induced DNA laddering that was attributed to an unidentified nuclease activity located in the IMS (Balk et al., 2003). It is, therefore, plausible that currently unknown mitochondrial proteins, other than cyt *c*, are involved in the regulation of plant PCD, and, hence, the mitochondrial role in plant cell death processes requires further investigation. In addition, chloroplasts have also been suggested to mediate PCD pathway(s) unique to plants, acting either downstream of mitochondria or in parallel with the mitochondrial signaling (van Aken and van Breusegem, 2015), with their involvement well supported by experimental evidence (e.g., Gan and Amasino, 1997; Samuilov et al., 2003; Doyle et al., 2010; Ambastha et al., 2015; Woodson, 2022).

PCD is often limited to small groups of dying cells buried in living tissue and, therefore, difficult to access, monitor, and sample, so there are often logistical challenges to studying the regulation of this process in whole plants (Reape and McCabe, 2008). In contrast, cell suspension cultures provided a useful model for studying PCD in species such as *A. thaliana* (Reape et al., 2015), carrot (McCabe et al., 1997), tobacco (Zhang et al., 2012), or wheat (Rezaei et al., 2013), as they enable precise monitoring of PCD rates induced by a range of stress treatments, as well as chemical manipulation of this process, and hence offer an unprecedented opportunity to specifically sample plant cells undergoing PCD. Indeed, plant suspension cells have been previously used to study plant PCD regulation at both transcriptome (Swidzinski et al., 2002) and proteome (Swidzinski et al., 2004) levels and to characterize plant PCD markers such as protoplast shrinkage (Kacprzyk et al., 2017). There are also well-established protocols for subcellular fractionation of plant suspension cells, allowing the isolation of relatively large volumes of intact mitochondria suitable for protein work (Reape et al., 2015). Previously, these characteristics of the cell suspension model facilitated research investigating the release of cyt *c* into cytosol during PCD induced by different types of stimuli (Malerba and Cerana, 2021) and studies of the mitochondrion-nucleus cross-talk during response to heat stress (HS) (Rikhvanov et al., 2007). Here, we used the state-of-the-art mass spectrometry (MS) to characterize the early, PCD-associated proteome changes in cytosolic and mitochondrial fractions from *A. thaliana* suspension cells. Cells were subjected to HS (10 min, 54°C) for induction of PCD. To facilitate identification of proteome changes specific to the PCD pathway, we used lanthanum chloride (LaCl_3_), a calcium channel blocker (Evans, 1990), previously reported to inhibit hallmark features of PCD such as early DNA fragmentation and protoplast shrinkage when applied before or even during early stress treatment (McCabe et al., 1997; Kacprzyk et al., 2017). Analysis of generated datasets deepened our understanding of events associated with plant PCD through identification of proteins translocated from mitochondria into cytosol upon stress insult, as well as changes in abundance of proteins occurring in the cytosolic fraction. Furthermore, Western blot (WB) experiments underscored the release of mitochondrial heat shock protein 60 (HSP60) into cytosol occurring in response to PCD inducing, but not sublethal level of HS, that may indicate its cytosolic role in plant PCD regulation.

## 2 Material and methods

### 2.1 Cell suspension cultures and growth conditions

*Arabidopsis thaliana* [ecotypes Landsberg *erecta* (Ler) and Columbia-0 (Col-0)] cell suspension cultures were grown as described previously (May and Leaver, 1993; Hogg et al., 2011). Cell suspension cultures were maintained under a 16-h light (~45 μmol m^−2^ s^−1^)/8-h dark in 250-ml Erlenmeyer flasks on an orbital shaker at 110 revolutions per minute (rpm) in a controlled environment room (22°C) and subcultured weekly by transferring 10 ml of cells into 100 ml of fresh growth medium. Seven-day-old, dark-grown cells were used for the cellular fractionation experiments.

### 2.2 PCD assay

Cell viability was assessed using fluorescein diacetate (FDA; Sigma-Aldrich) staining as previously described (Reape and McCabe, 2008; Hogg et al., 2011). Cells positive for FDA staining were scored as alive, and dead cells showing no fluorescence were categorized as PCD, if they displayed a retracted cytoplasm, or as necrotic, if they had no protoplast retraction. Three biological replicates per treatment with each three technical replicates of at least 200 cells were scored per data point.

### 2.3 Heat stress treatment

Prior to experiments, flasks of 100 ml of 7-day-old dark-grown suspension cells were examined for viability. The flasks showing >95% viability were pooled together to ensure use of homogenous biological material for experiments. Heat treatments were performed in Grant OSL200 water bath set to 85 rpm using 100 ml of aliquots of suspension cells in 250-ml Erlenmeyer flasks (PCD induction, 54°C for 10 min; sublethal stress, 33°C for 10 min). To inhibit PCD, cells were pre-incubated with 750 µM LaCl_3_ (Acros Organics) for 30 min in the dark prior to HS treatment.

### 2.4 Isolation of mitochondrial and cytosolic fractions

Mitochondrial and cytosolic fractions were isolated as previously described (Reape et al., 2015) using 100 ml of cell suspension culture per treatment. The heat-treated samples were cooled on ice and processed within 10 min of heat treatment. Cells that were not subjected to heat treatment required homogenization using glass beads (diameter of 425–600 μm; Sigma-Aldrich) in a mortar and pestle three times for 4 min to achieve >80% cell disruption, whereas, for heat-treated samples, two times for 4 min were sufficient. The homogenization buffer was changed after every filtration. The homogenized cells were centrifuged at 2,000g for 10 min (4°C) to remove cell debris and the glass beads, and the supernatant was subjected to centrifugation at 13,000g for 15 min (4°C). The resulting supernatant, 250 µl (cytosolic fraction) was mixed with 750 µl of protein lysis buffer (6 M urea, Lennox Laboratory Supplies; 2 M thiourea, Fisher Chemical; supplemented with protease inhibitor cocktail, Roche, cOmplete™ mini), whereas the pellet (crude mitochondrial fraction) was further processed following the protocol from Reape et al. (2015). The purified mitochondrial pellets were then resuspended in 200 µl of protein lysis buffer. Fractions were stored at −80°C until subsequent proteomic/WB analyses.

### 2.5 Protein extraction and purification

Proteins were prepared for MS analysis as previously described (Eitle et al., 2019) with the following modifications. The samples were not sonicated, and only mitochondrial fractions were homogenized with a motorized pestle for 10 s. Protein concentration for each sample was determined using a Qubit fluorometer (Invitrogen) and the Quant-iT protein assay kit (ThermoFisher) according to the manufacturer’s instructions. Proteins (100 µg per sample) were purified using the 2-D Clean-up Kit (GE Healthcare) as per the manual (procedure A). After in-solution digestion, the peptides were purified using ThermoScientific C18 spin columns, and the final peptide eluant of 90 µl was dried using a SpeedyVac and stored at 4°C.

### 2.6 Mass spectrometry and functional annotation

Four biological replicates for each group (total, n = 32) were subjected to high resolution MS. Samples were removed from 4°C storage and equilibrated to room temperature for 10 min before adding the Q Exactive Loading Buffer (2% acetonitrile (ACN) and 0.05% trifluoroacetic acid (TFA) in ddH_2_O) to final peptides concentration of 0.5 µg/µl. The tubes were briefly vortexed and sonicated for 3 min to resuspend the proteins. Afterward, the samples were centrifuged for 5 min at 19,000g, and the supernatant was transferred into the mass spec vials (Thermo Scientific Waltham, USA). The same specification and setup were used as previously described (Eitle et al., 2019). MaxQuant v1.6.17.0 (Tyanova et al., 2016, www.maxquant.org/) was used to perform protein identification and label-free quantification (LFQ) normalization. The generated MS/MS data were searched against both the predicted proteome derived from the *Arabidopsis thaliana* TAIR11 reference genome (Berardini et al., 2015; https://www.arabidopsis.org/, accessed September 2021, containing 48,231 peptide sequences) and a contaminant sequence set supplied by MaxQuant, using the Andromeda algorithm (Hubner et al., 2010). The MS proteomics data and MaxQuant search output files have been deposited to the ProteomeXchange Consortium (Côté et al., 2012) *via* the PRIDE partner repository with the dataset identifier PXD040584. After filtering for environmental protein contaminants, the LFQ intensities were log_2_-transformed, and the samples were grouped on the basis of their fraction and treatment (e.g., cytosolic control). To visualize the differences between the treatments, the dataset was prepared for analysis with a principal component analysis (PCA). Only proteins that were found in all replicates of at least one of the groups were retained, and a data imputation step was conducted to replace missing values (NaN) with intensities that simulate proteins with low abundance. The PCA was carried out using RStudio v3.6.2 (R Development Core Team, 2014; https://www.R-project.org/) and visualized with ggplot2 (Wickham, 2009). Volcano plots comparing two groups were generated in Perseus, by plotting the −Log P-values on the *y*-axis and the log_2_ fold–transformed difference between the groups on the *x*-axis. The false discovery rate (FDR) cutoff was set to 0.05, and a minimal fold change cut off used was 1.5 [s0 = log_2_(1.5)].

### 2.7 Bioinformatics

Subcellular protein localization was predicted using the quick search tool from SUBA4 (Hooper et al., 2014; Hooper et al., 2017; https://suba.live) and MuLocDeep (Zhang et al., 2018; Jiang et al., 2021; Jiang et al., 2023; https://www.mu-loc.org/). Fasta files as input for the MuLocDeep were obtained from TAIR (Berardini et al., 2015; www.arabidopsis.org/) with the following options selected: “Araport11 protein sequence” and “get one sequence per locus”. PCA of generated proteomes was carried out using RStudio v3.6.2 (R Development Core Team, 2014; https://rstudio.com) and visualized with ggplot2 (Wickham, 2009). Gene annotation and pathway enrichment analysis were performed with Metascape (Zhou et al., 2019; https://metascape.org). Functional analysis was carried out with String (version 11.5; Szklarczyk et al., 2021; https://string-db.org).

### 2.8 Comparison of proteins released from mouse and *A. thaliana* mitochondria

To compare mammalian and plant mitochondrial proteins involved in cell death, a list of proteins that were released from mitochondria undergoing permeability transition in mouse liver cells (Patterson et al., 2000) was compared to list of proteins released from *Arabidopsis* mitochondria in this study. The annotation-based analysis was performed by comparing protein names. For sequence-based comparison, FASTA sequences of the mammalian proteins listed by (Patterson et al., 2000) were obtained from UniProt (UniProt Consortium, 2021) or The National Center for Biotechnology Information (NCBI) (Sayers et al., 2022). These sequences were blasted on NCBI (blastp) with *Arabidopsis thaliana* set as target organism. The top three results are listed in **Supplementary Table 5**.

### 2.9 Western blot

Cytosolic and mitochondrial fractions were thawed on ice and mixed. Samples (10 µg of protein) were combined with 4× Laemmli buffer (Bio-Rad) and 5% β-mercaptoethanol (Sigma-Aldrich). All samples were briefly vortexed, spun down, and heat-treated for 5 min at 95°C before loading on precast 8%–16% polyacrylamide gels (Bio-Rad) in a tank buffer [25 mM Tris (Fisher Bioreagents), 192 mM glycine (Fisher Bioreagents), and 0.1% sodium dodecyl sulfate (SDS) (VWR Life Science)]. After the electrophoresis, proteins were transferred onto 0.2 µM nitrocellulose membrane (Bio-Rad, 20 V for 7 min or 5 min for cytochrome c), and the transfer was validated *via* Ponceau staining (Sigma-Aldrich). The blots were rinsed twice and washed three times for 5 min with Tris-Glycine-Tween-20 (TGT) buffer under gentle shaking [25 mM Tris, 1.92 M Glycine, and 0.2% Tween 20 (Thermo Scientific)]. The membranes were blocked for 1 h with 5% skimmed milk powder (Marvel) in TGT and, subsequently, incubated with primary anti-body overnight. Antibodies and respective concentrations used are as follows: anti-mouse HSP60 (LK2) (ENZO ADI-SPA-807E), 1:2,000; anti-rabbit cytosolic HSP70 (Agrisera AS08 371), 1:5,000, anti-rabbit IDH2 (Agrisera AS06 203A), 1:3,000; and anti-mouse mitochondrial HSP70 (Agrisera AS08 347), 1:3,000. Blots were rinsed twice and washed three times for 10 min with TGT buffer, followed by a 1-h incubation with anti-mouse (Biosciences Ltd., 32430) or anti-rabbit antibody (Biosciences Ltd., 656120) at a 1:2,000 dilution. Anti-mtHSP70 was incubated for 2 h. After another two rinses and three 10-min washes, four washes for anti-ctHSP70, chemiluminescent was detected with Western Lightning Plus ECL substrate (PerkinElmer), and images were acquired with the LAS-4000 Image Analyzer (Fujifilm). Exposure times varied per anti-body between 2 and 8 min, as indicated per blot (**Supplementary Figure S1**). Densitometric analyses of the chemiluminescent were performed using GelAnalyzer 19.1 (www.gelanalyzer.com) by Istvan Lazar Jr., PhD, and Istvan Lazar Sr., PhD, CSc.

## 3 Results

### 3.1 Effect of heat stress and lanthanum chloride on PCD rates in *A. thaliana* suspension cells

**Figure 1:**
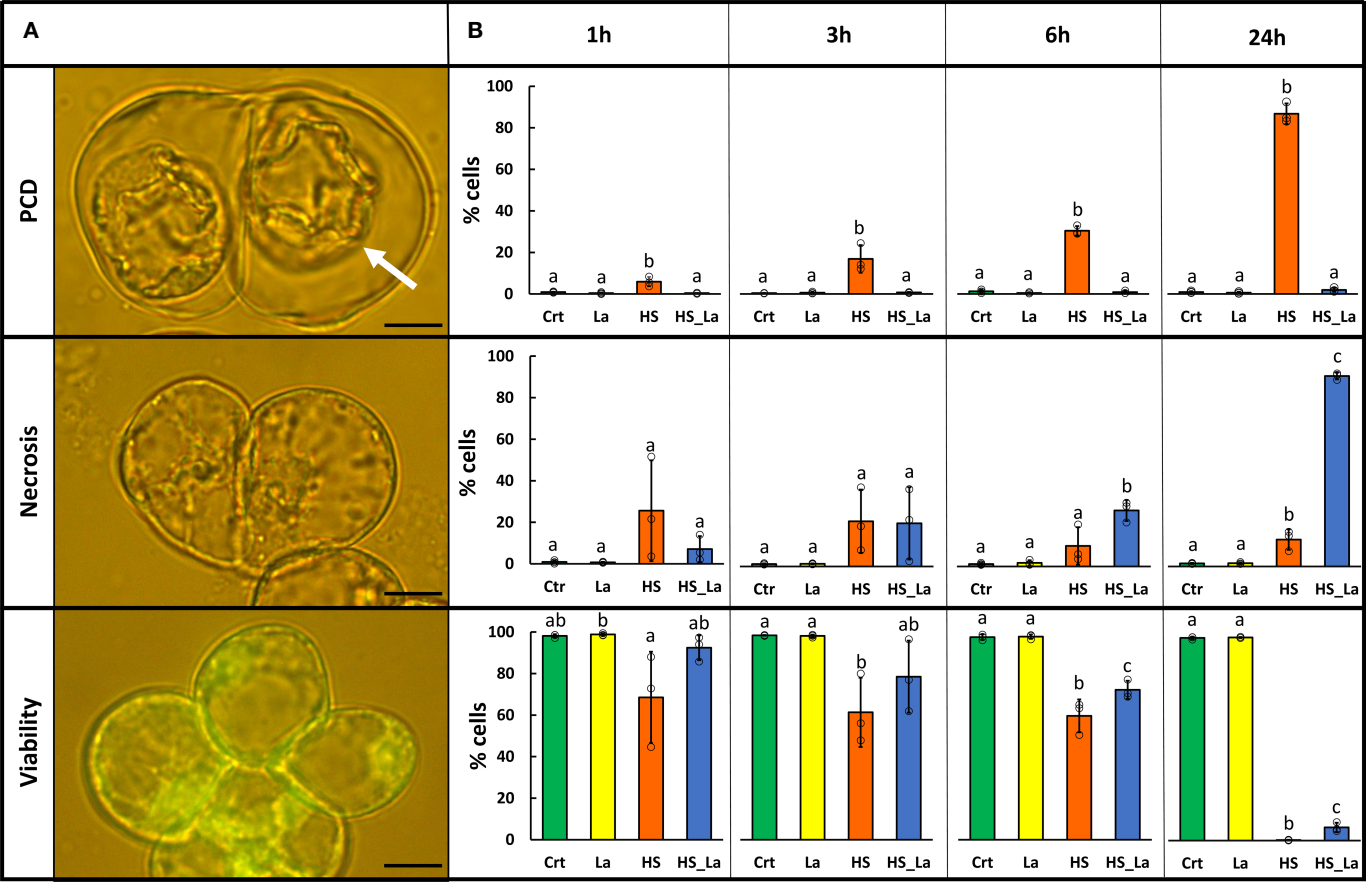
LaCl_3_ modulates the rates of PCD induced by heat stress (HS) in *A. thaliana* suspension cells (Ler ecotype). **(A)** FDA-stained cells were categorized on the basis of their morphology as previously described (Reape and McCabe, 2008). Cells that died through PCD exhibit no fluorescence and present hallmark morphology with easily identifiable retraction of the protoplast (white arrow). Scale bar, 10 µm. **(B)** Rates of PCD, necrosis, and viability 1, 3, 6, and 24 h following HS (54°C, 10 min) with and without 750 µM LaCl_3_. Bars represent means of three experiments ± SD, where each sample has been scored three times (200 cells examined each time). Individual data points are indicated. Following ANOVA, differences between treatments at each time point were analyzed using Tukey honestly significant difference (HSD) *post-hoc* test. Means labeled with different letters are statistically different at 0.05 level. Crt, control; La, LaCl_3_; HS, heat stress; HS_La, heat stress in presence of LaCl_3_.

HS (54°C, 10 min) was applied to induce PCD in *A. thaliana* suspension cells (ecotype *Landsberg erecta*, Ler), and, 30 min pre-treatment with extracellular calcium channel blocker, lanthanum chloride (750 µM LaCl_3_) was used to inhibit it, as previously described (Kacprzyk et al., 2017). The resulting rates of PCD, necrosis, and viability (**Figure 1A**) were determined 1, 3, 6, and 24 h after HS (**Figure 1B**). Twenty-four hours following HS, 87% of cells underwent PCD, manifesting as loss of FDA fluorescence and development of hallmark PCD morphology: shrinkage of the protoplast away from the cell wall. As previously observed (McCabe et al., 1997; Kacprzyk et al., 2017), the presence of LaCl_3_ caused complete inhibition of PCD, and this loss of a regulated cell death pathway instead led to damaged cells dying *via* necrosis, considered to be an uncontrolled cell death mode. Treatment with LaCl_3_ also significantly delayed the loss of viability following the HS, although this effect was less pronounced. There was no significant effect of LaCl_3_ on rates of PCD, necrosis, and viability in cells that were not subjected to HS. Subsequently, we used this experimental system for generation of proteomic datasets characterizing changes in protein abundance and localization associated with the early stages of PCD induction. The cells were subjected to HS in the presence and absence of 750 µM LaCl_3_, as described above, and the cellular fractionation protocol was started directly (within 10 min) following the HS. Subsequently, MS-based proteomics was performed on the purified fractions to generate mitochondrial and cytosolic proteomes.

### 3.2 Subcellular fractionation protocol results in enrichment of mitochondrial proteins in the isolated mitochondrial fraction

The proteomes of mitochondrial and cytosolic fractions of control (untreated) cells were analyzed to evaluate the purity of isolated mitochondria. A total of 2,674 proteins were detected across both fractions, of which 1,062 were unique to the cytosolic fraction and 688 were unique to the mitochondrial fraction (**Table 1**; **Supplementary Table S1**). Subsequently, we compared our dataset to a previously published *A. thaliana* mitochondrial proteome (Jiang et al., 2021). This revealed that 89 % (612) of proteins uniquely detected here in mitochondrial fraction were previously associated with this organelle, in contrast to only 14% (153) of unique cytosolic proteins (**Table 1**). Furthermore, the cellular localization of the remaining 76 proteins unique to the mitochondrial fraction was analyzed using SUBA4 (Hooper et al., 2014; Hooper et al., 2017) and MuLocDeep (Jiang et al., 2021; Jiang et al., 2023) (**Supplementary Tables S1**, **S2**). Consensus localization predicted by the SUBcellular location database for Arabidopsis proteins version 4 (SUBA4) for these proteins was mitochondrial (33 proteins) and plastidic (22 proteins) with the remaining proteins distributed across other cellular compartments (**Supplementary Table S2A**). Similarly, MuLocDeep annotated most of the investigated proteins as mitochondrial (33 proteins) or plastidic (19 proteins) (**Supplementary Table S2B**). Collectively, these results confirmed that mitochondrial proteins were enriched in the mitochondrial fraction isolated from *A. thaliana* suspension cells and, this way, validated the subcellular fractionation protocol employed in this study.

**Table 1:** MS profiling confirms enrichment in mitochondrial proteins in isolated mitochondrial fraction.

A	Cytosolic fraction	Unique cytosolic proteins	Mitochondrial fraction	Unique mitochondrial proteins
Total proteins identified	1,986	1,062	1,612	688
Proteins included in *Arabidopsis* mitoproteome by Jiang et al. (2021)	754	153	1,213	612
	38%	14%	75%	89%

Proteins detected in cytosolic and mitochondrial fractions from control (untreated) *A. thaliana* suspension cells, compared to previously published *Arabidopsis* mitoproteome by Jiang et al. (2021).

### 3.3 The effect of PCD-inducing heat stress and lanthanum chloride on mitochondrial and cytosolic proteomes

**Figure 2:**
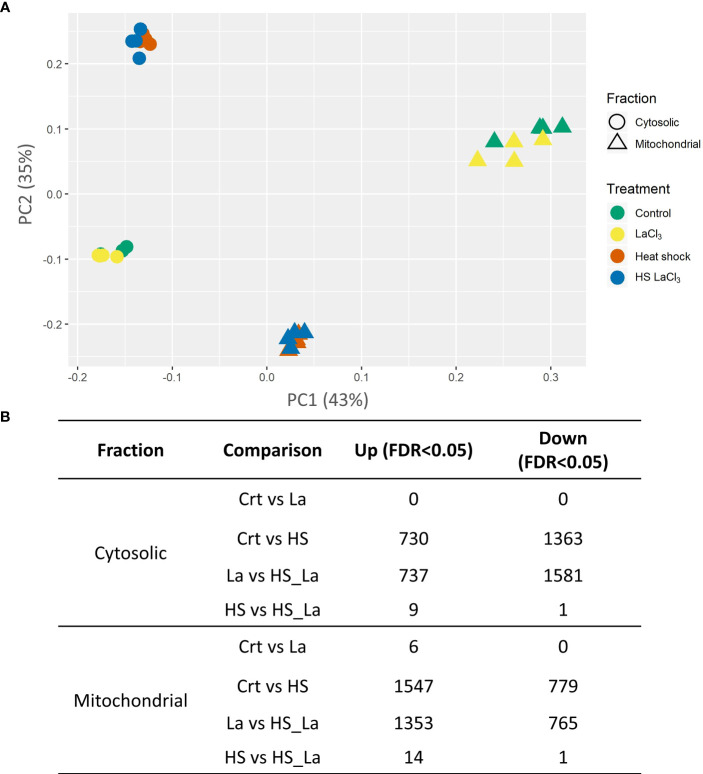
Effect of PCD-inducing heat stress and lanthanum chloride pre-treatment on the proteome profiles of mitochondrial and cytosolic fractions from *A. thaliana* suspension cells. **(A)** PCA plot of analyzed protein profiles (n = 4). **(B)** Number of proteins differing in abundance (minimum, 1.5-fold change) between the indicated sample types at false discovery rate (FDR) cutoff of 0.05 and 0.1. Total proteins detected across all samples: 2,840; individual proteins are listed in **Supplementary Table S3**.

PCA of the generated proteome datasets highlighted distinct protein compositions of cytosolic and mitochondrial fractions, with extensive changes induced by the PCD-inducing HS treatment (**Figure 2A**). In contrast, the effect of lanthanum chloride on the global proteome profiles of both fractions appeared marginal. This was confirmed by the subsequent quantitative analysis of relative protein abundances (**Figure 2B**; **Supplementary Table 3**) that revealed changes in abundance for the majority of detected proteins following the HS. Of 2,840 proteins detected across all samples, 2,093 proteins in the cytosolic and 2,326 proteins in the mitochondrial fraction changed their abundance in response to HS at least 1.5-fold at 0.05 significance level (**Supplementary Table S3**). The changes induced by LaCl_3_ treatment alone were small in comparison, with no proteins changing their abundance in the cytosolic fraction, and only six proteins being upregulated in the mitochondrial fraction (**Figure 2B**). Comparison of samples heat-treated in the absence and presence of LaCl_3_ (HS *vs*. HSLaCl_3_) also identified only a small number of proteins differing in abundance: 10 (cytosolic fraction) and 15 (mitochondrial fraction).

**Figure 3:**
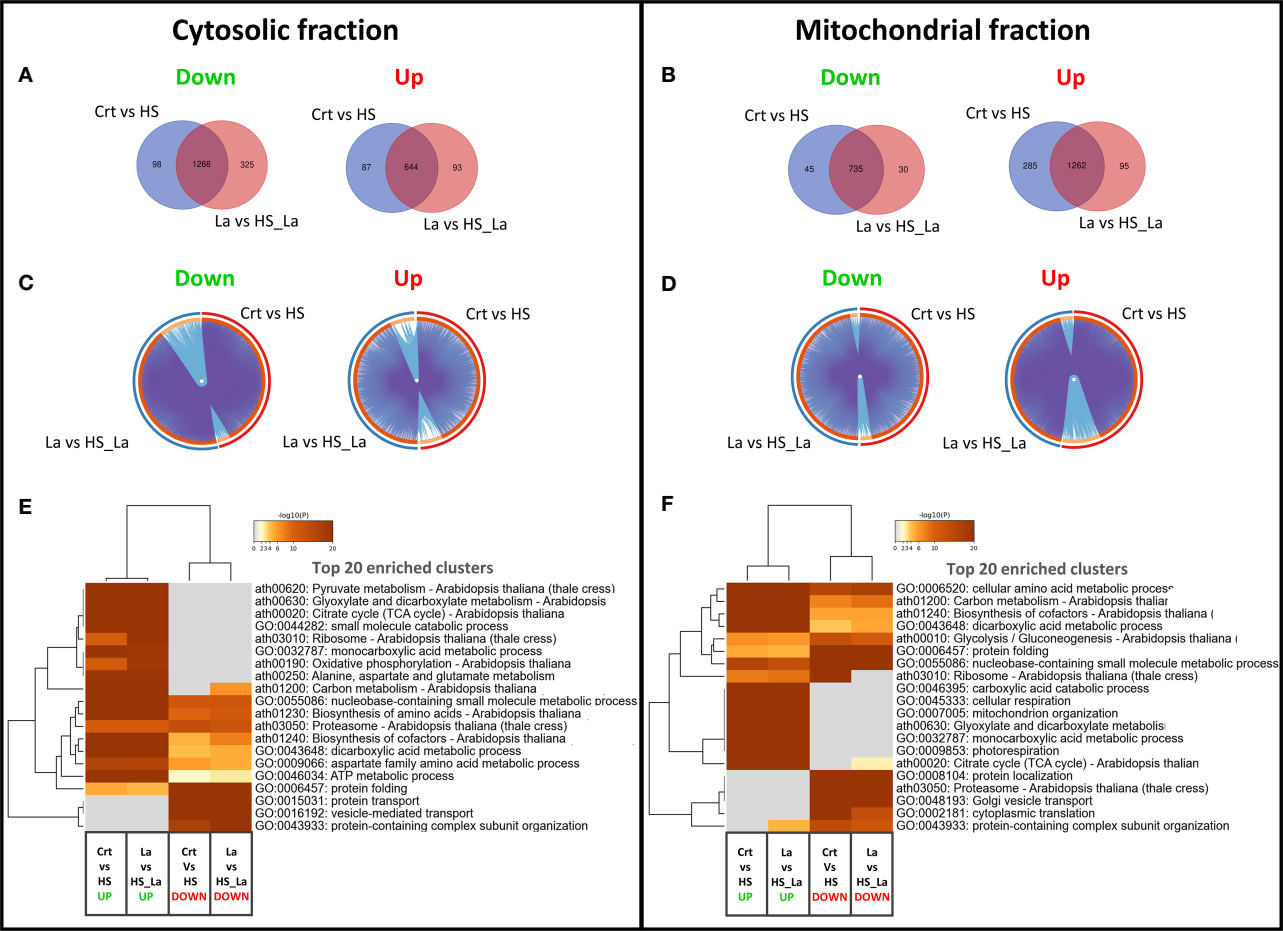
Changes in proteome profiles of cytosolic and mitochondrial fractions induced by heat treatment. **(A, B)** Venn diagrams for proteins down- and upregulated in response to HS, in the presence and absence of LaCl_3_ in cytosolic and mitochondrial fractions. **(C, D)** The Circos plots compare the overlap between proteins down- and upregulated by HS in the presence of LaCl_3_ in cytosolic and mitochondrial fractions, respectively. The outside arc indicates the protein list identity, the orange color on the inside arc represents proteins that appear in multiple lists, and the light orange color represents proteins unique to that list. Purple lines link the genes shared by both lists, whereas the blue lines link the proteins falling into the same enriched ontology term. **(E, F)** Clustered heatmaps presenting 20 top enriched clusters, and 20 most selective enriched clusters, as well as their enrichment patterns for cytosolic and mitochondrial fractions. The heatmaps are colored by the significance (log p-value) of enrichment. The minimum enrichment factor was set to 2 with p-value cutoff of 0.01.

We also investigated the effect of LaCl_3_ on the HS-induced proteome changes in the cytosolic and mitochondrial fractions (**Figure 3**). There was a notable overlap between proteins up- and downregulated in response to HS in the presence and absence of LaCl_3_ in both fractions (**Figures 3A, B**). As the similarities and differences between proteomic datasets may be more readily recognized at the pathway level than by comparing lists of individual proteins, we also performed a functional enrichment analysis using Metascape (Zhou et al., 2019). As highlighted by the Circos plots (**Figures 3C, D**), the functional overlap among proteins up/downregulated in the presence and absence of LaCl_3_ in both fractions (indicated by the blue links) exceeds the overlap between the respective protein lists (indicated by purple links), further underscoring the limited effect of LaCl_3_ on global proteomic changes induced by HS. The top 20 enriched clusters were largely unchanged in the presence of LaCl_3_ (**Figures 3E, F**). However, for the proteins upregulated in the mitochondrial fraction, HS caused enrichment of cluster of Gene Ontology (GO) terms related to the ribosome (ath03010: Ribosome – *Arabidopsis thaliana*) in the absence but not in the presence of LaCl_3_.

### 3.4 Identification of candidate proteins involved in plant PCD

Release of mitochondrial proteins into the cytosol is a key event of the apoptotic pathway in animal cells (Bock and Tait, 2020). In the present study, 288 proteins were upregulated in the cytosol, but downregulated in the mitochondrial fraction following the HS (**Supplementary Table S4A**), which may suggest their translocation into the cytosol in response to PCD-inducing stimuli. The majority of the proteins had mitochondrial (125 proteins), plastidic (107), or peroxisomal (27) localization according to SUBA4 (**Supplementary Table S4A**). It needs to be noted that, although proteins from different cellular compartments may copurify with the mitochondrial fraction as an artifact of cellular fractionation protocol, they can be also localized to multiple organelles (e.g., to both mitochondria and plastids), which is not always registered by protein localization databases (Jiang et al., 2021). **Supplementary Table S4A** provides a summary of available experimental evidence for their localization (based on MS and/or studies using fluorescent protein–based visualizations).

Of the 125 proteins annotated as mitochondrial, 113, including cyt *c*, showed HS-induced downregulation in mitochondrial fraction greater than that observed for the matrix protein isocitrate dehydrogenase [NADP(+)] type 2 (IDH2; AT2G17130, log2FC = −2.09762) that is frequently used as mitochondrial structural marker (Rikhvanov et al., 2007; Lopez-Huertas and del Río, 2014; Lee et al., 2019; **Supplementary Table S4A**, highlighted in yellow), indicating that, although populations of mitochondria lose structural integrity and burst early after the PCD-inducing treatment, the controlled release of specific proteins, such as cyt *c*, is also observed. This group of proteins also included nucleoside diphosphate kinase (NDPK3) with nuclease activity specific to structured supercoiled nucleic acids that was previously hypothesized to play a role in PCD-associated DNA degradation after its translocation to the nucleus (Hammargren et al., 2007). Hexokinase I (HX-1) was also released, which might be significant considering the multiple lines of evidence, suggesting that plant mitochondrial hexokinases can modulate PCD through interactions with voltage-dependent anion channel (VDAC) proteins in the OMM (Kim et al., 2006; Godbole et al., 2013).

When HS was performed in the presence of the PCD inhibitor LaCl_3_, only 18 (6.3%) of the 288 proteins translocated from the mitochondrial fraction were not downregulated in the mitochondrial fraction (**Table S4A**, highlighted in green). None of them were annotated as mitochondrial by SUBA4, with 12 proteins having plastidic, two peroxisomal, and four cytosolic localizations. This suggests that LaCl_3_ treatment had no effect on the stress-induced release of mitochondrial proteins into cytosol but could have partially ameliorated stress-induced degradation of organelles copurifying with the mitochondrial fraction (plastids and peroxisomes). The proteins released from the mitochondria of *A. thaliana* after PCD-inducing stimuli were also compared to the proteins released from mouse mitochondria following the chemically induced permeability transition (Patterson et al., 2000; **Supplementary Table S5**). Briefly, putative *Arabidopsis* homologs were identified for 69 of the 76 known proteins listed by Patterson et al. (2000) based on sequence similarity and/or annotation as detailed in Materials and Methods (**Supplementary Table S5**) and compared to proteins translocated from mitochondrial to cytosolic fraction in the present study (**Table S4**). For 52 (>75%) proteins released from mouse mitochondria, at least one of the identified putative *Arabidopsis* homologs was also released from the mitochondrial fraction following the HS. A total of 74 proteins that were translocated from plant mitochondria into cytosol were putative homologs of proteins released from this organelle in mice during permeability transition (Patterson et al., 2000). We also performed a functional enrichment on the list of the remaining 442 proteins upregulated in the cytosol following the heat shock but not downregulated in mitochondrial fraction (**Supplementary Table S4B**). String-DB (version 11.5, Szklarczyk et al., 2021) analysis highlighted the enrichment of terms that may be linked not only to the PCD-associated degradation of cellular content, such as peptidase activity (GO:0008233, 34 proteins) and hydrolase activity (G0:0016787, 82 proteins), but also to the activation of pro-survival signaling, such as antioxidant activity (GO:0016209, 14 proteins) (**Supplementary Material File SM1**). Most of these 442 proteins upregulated in the cytosolic fraction were annotated as cytosolic (211), plastidic (71), nuclear (48), or, interestingly, extracellular (or vacuolar) (13) (SUBA4, **Table S4B**). Many of them were previously identified as cell death and stress response modulators in plants (**Table S4B**, highlighted in yellow). The observed upregulation of 33 extracellular proteins in the cytosolic fraction early after PCD-inducing treatment is of particular interest, considering previous studies reporting proteins normally residing in extracellular space being imported back into cytosol to play a role in plant PCD (Chichkova et al., 2010; Williams et al., 2015). String-DB analysis highlighted term “Hydrolase activity” GO:0016787 as being significantly enriched with 16 members of this group including six proteins with protease activity (subtilisin-like protease SBT2.2, AT4G20430; AT4G12910, scpl20; AT1G15000, scpl50; AT5G67360.1, ARA12; AT2G22970.3, SCPL11; AT4G36195.3, AT4G36195) and one ribonuclease (AT2G02990, RNS1). Notably, upregulation of 138 (31%) of the 442 proteins in the cytosolic fraction (SUBA4 annotation: 62 cytosolic, 18 plastidic, 23 nuclear, nine extracellular, and seven vacuolar) was not observed when HS was performed in the presence of PCD inhibitor LaCl_3_ (**Supplementary Table S4B**, highlighted in green), suggesting that the changes in their cytosolic abundance may be linked to the PCD process.

### 3.5 Large-scale downregulation of proteins in the cytosolic fraction following the PCD-inducing HS

In the cytosolic fraction, 1,362 proteins were downregulated following the PCD-inducing HS (**Supplementary Table S6A**). This indicates that degradation of the cell content may start early after the stress insult and can be linked to the observed upregulation of proteins with hydrolytic activity in this fraction. The targeted degradation of pro-survival proteins may also be a key event in the initiation and/or execution of the plant PCD pathway. Indeed, the list of proteins undergoing downregulation in the cytosolic fraction included Tudor staphylococcal nuclease 1 and 2 (TSN1 and TSN2). TSN sustains cell viability, and cleavage of TSN appears an evolutionary conserved element of the cell death pathway in both plants and animals (Gutierrez-Beltran et al., 2016). However, 1,026 of the 1,362 proteins downregulated in the cytosolic fraction following HS were also simultaneously upregulated in the mitochondrial fraction (**Supplementary Table S6A**). The observed upregulation of a large number of proteins in the mitochondrial fraction following the HS can at least partially result from stress-induced aggregation of cytoplasmic proteins that further copurify with the mitochondria during the subcellular fractionation protocol, such as stress granules (SGs) and other membraneless cytoplasmic protein assemblies. Although there are limited data on the proteome composition of the plant SGs, out of the 118 SGs localized proteins recently identified in *Arabidopsis* (Kosmacz et al., 2019), 75 were upregulated in the mitochondrial fraction after the HS, including SG markers such as RNA BINDING PROTEIN 47 A (RBP47), RBP47B, OLIGOURIDYLATE-BINDING PROTEIN 1C (AtUBP1c, AT3G14100), and POLY-A BINDING PROTEIN 2 (PABP2) (Sajeev et al., 2022). Furthermore, Kosmacz et al. (2019) reported cross contamination of purified SGs with mitochondrial and plastidic proteins, and SGs were reported to physically interact with mitochondria in animal cells to regulate metabolic remodeling (Amen et al., 2021). Therefore, formation of SGs, as well as other cytosolic protein aggregates, is a potential explanation for the observed upregulation of proteins in the mitochondrial fraction.

String-DB analysis revealed significant enrichment of many terms related to cytoprotective and stress response pathways (**Supplementary Material File SM2**). Examples include terms related to stress response (e.g., GO:0006950 Response to stress, 252 proteins; GO:0033554 Cellular response to stress, 109 proteins; GO:0034976 Response to endoplasmic reticulum stress, 21 proteins; and GO:0009266 Response to temperature stimulus, 69 proteins). Other enriched terms are related to proteasome function and structure [e.g., GO:0043161 Proteasome-mediated ubiquitin–dependent protein catabolic process (28 proteins), GO:0043248 Proteasome assembly (six proteins), GO:0005838 Proteasome regulatory particle (23 proteins), GO:0000502 Proteasome complex (25 proteins), and GO:0031597 Cytosolic proteasome complex (nine proteins)], DNA repair [e.g., GO:0006281 DNA repair (35 proteins), GO:0000727 Double-strand break repair *via* break-induced replication (six proteins), GO:0061077 Chaperone-mediated protein folding (14 proteins), GO:0044183 Protein folding chaperone (nine proteins), GO:0051085 Chaperone cofactor-dependent protein refolding (11 proteins), and GO:0051131 Chaperone-mediated protein complex assembly (four proteins)], and heat shock proteins [e.g., GO:0031072 Heat shock protein binding (15 proteins), IPR001404 Heat shock protein Hsp90 family (six proteins), and IPR013126 Heat shock protein 70 family (nine proteins)]. Moreover, in line with the hypothesis that the observed downregulation of cytosolic proteins could at least partly be due to their sequestration into SGs, terms related to SG formation [e.g., GO:0010494 Cytoplasmic SG (10 proteins) and GO:0034063 SG assembly (four proteins)] and SG function in translational control [e.g., GO:0006417 Regulation of translation (50 proteins) and GO:0006412 Translation (80 proteins)] were significantly enriched. Furthermore, to identify which of these stress-induced changes might be particularly relevant in the context of cell death regulation, we examined the effect of PCD inhibitor and calcium channel blocker LaCl3 on heat-induced downregulation of proteins from the cytosolic fraction. The presence of LaCl3 inhibited downregulation of 109 proteins (**Supplementary Table 6a**, highlighted in green). STRING-DB analysis highlights a cluster of these proteins enriching terms GO:0006412 Translation (nine proteins), GO:0010467 Gene expression (17 proteins), and Kyoto Encyclopedia of Genes and Genomes (KEGG) pathway ath03010 Ribosome (12 proteins) (**Supplementary Materials File SM3**). Only one protein, BCL-2–associated athanogene 7 (BAG7, AT5G62390.1), showed an opposite direction of regulation in the cytosolic fraction due to LaCl3 treatment. BAG7 was downregulated by HS in the cytosolic fraction but upregulated if HS was performed in the presence of LaCl3. This may suggest one of the mechanisms underlying the pro-survival effect of LaCl3, as BAG7 is an essential element of unfolded protein response and its knockout mutants are more sensitive to heat and cold (Williams et al., 2010). Moreover, several other proteins that were downregulated by HS in the cytosolic fraction, but not in the presence of LaCl3, were also previously described in context of cell death and stress responses (**Supplementary Table 6b**, highlighted in red). This includes UP-FRAMESHIFT1 (UPF1)/LOW-LEVEL BETA-AMYLASE 1 (LBA1), required for nonsense-mediated mRNA that, under normal conditions suppresses activation of plant immunity (Raxwal et al., 2020), as well as LYSOPL2, involved in tolerance to oxidative stress (Gao et al., 2010).

### 3.6 Changes in abundance of heat shock proteins

**Figure 4:**
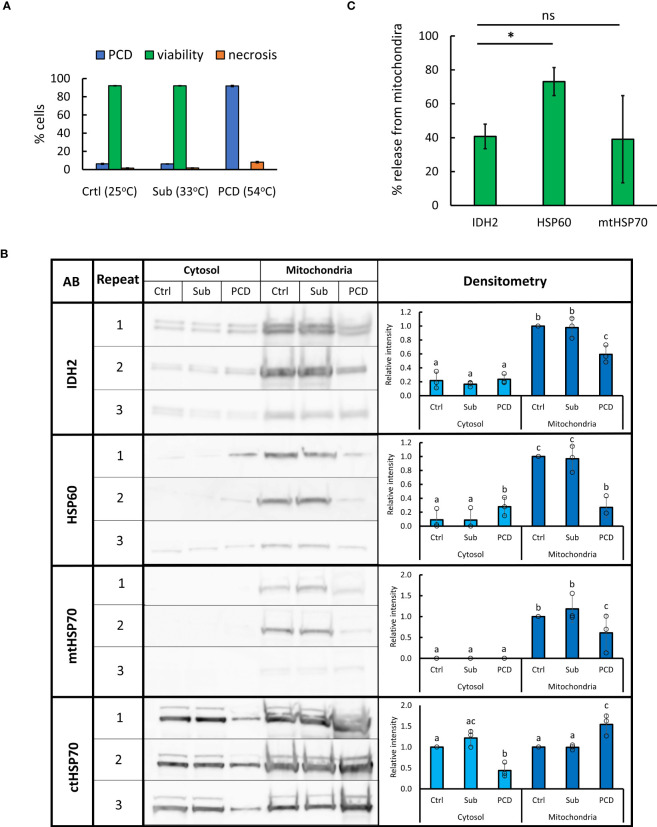
Changes in abundance of selected HSPs in mitochondrial and cytosolic fractions following sub-lethal and PCD-inducing heat treatment. **(A)** *Arabidopsis* cell suspension culture (ecotype Col-0) was subjected to 10 min of control (25°C), sublethal (33°C), or PCD-inducing (54°C) temperature. Bars represent mean of three experiments ± SD for rates of PCD, necrosis, and viability 24 h following heat treatment. **(B)** For WB, 10 µg of proteins from cytosolic and mitochondrial fractions, isolated directly after heat treatment, were loaded per lane. Densitometry analysis was performed using GelAnalyzer. Bars represent mean signal intensity (± SD) relative to signal of mitochondrial control fraction (IDH2, HSP60, and mtHSP70) or control cytosolic fraction (ctHSP70). Means labeled with different letters are statistically different at 0.05 level (ANOVA, followed by Tukey HSD *post-hoc* test). AB, antibody; Sub, sublethal; Crtl, control. **(C)** Percentage of mitochondrial proteins released after PCD-inducing HS (54°C, 10 min). Bars represent mean of three experiments ± SEM, *p < 0.01 (paired Student’s t-test).

HSPs are molecular chaperones mediating a diverse range of processes including stress signaling; unfolded protein response; formation of SGs; protein translocation, targeting, and degradation; and PCD (Vierling, 1991; Kotak et al., 2007; Dogra et al., 2019; Verma et al., 2021). Analysis of the generated proteomes revealed that HSPs with consensus localization (SUBA4) in the cytosol generally decreased in abundance in the cytosolic fraction following the HS, with many of them showing simultaneous increase in the mitochondrial fraction (**Supplementary Table S7**). However, many of HSPs with consensus localization in the mitochondria, including HSP60s (AT3G23990, AT2G33210, and AT3G13860) and HSP70s (AT5G09590 and At4G32208) family members, demonstrated the opposite trend, with abundance decreasing in the mitochondrial fraction and going up in the cytosol after the HS, possibly caused by their release from the mitochondria during PCD. To ascertain whether these changes in HSP abundance are specific to PCD induction rather than the heat response in general, we subjected *A. thaliana* (Col0) suspension cells to PCD (10 min, 54°C) or sublethal (10 min, 33°C) stress treatment (**Figure 4A**). Sublethal level of stress was determined by identifying the highest HS temperature that does not result in viability drop below 90% within 24 h post-treatment. Subsequently, cytosolic and mitochondrial fractions were isolated directly following the treatment and WB used to identify changes in abundance of cytosolic and mitochondrial HSP70 as well as mitochondrial HSP60 and IDH2 (structural marker for this organelle (Rikhvanov et al., 2007; Lopez-Huertas and del Río, 2014; Lee et al., 2019) (**Figure 4B**). The results suggested that levels of IDH2, HSP60, and mtHSP70 decrease in the mitochondrial fraction in response to PCD-inducing, but not to sublethal, levels of heat. The decrease of cytosolic HSP70 was also observed only in the case of PCD-inducing treatment. The release of HSP60, but not mtHSP70, exceeded that observed for the mitochondrial matrix protein and structural marker IDH2 (**Figure 4C**). This further supports the controlled release of HSP60 from plant mitochondria occurring during PCD induction. Overall, the WB results were in agreement with MS-based observations and the use of second cell suspension culture of a different ecotype, which additionally validated the reported findings. Full images of immunoblots and respective exposure times are available in **Supplementary Figure S1**.

## 4 Discussion

We used an approach combining *Arabidopsis* cell suspension culture, a well-established model system for studying plant PCD (Swidzinski et al., 2002; Swidzinski et al., 2004; Reape et al., 2015), with cellular fractionation and the state-of-the art MS-based proteomics to obtain an in-depth profile of the proteomic changes associated with early stages of plant PCD induced by HS. In addition, we investigated the effect of the calcium channel blocker, LaCl_3_, on HS-induced changes in protein abundance. This approach enabled the identification of proteins specifically associated with the PCD process, as opposed to the general cellular stress response to heat.

### 4.1 Changes to mitochondrial proteome upon PCD induction

We first confirmed that the subcellular fractionation protocol results in enrichment of mitochondrial proteins in the isolated mitochondrial fraction and, therefore, is suitable for studying early mitochondrial proteome changes associated with plant PCD. This was achieved using a previously published *Arabidopsis* cell culture mitoproteome (Jiang et al., 2021), as well as protein localization database SUBA4 (Hooper et al., 2014; Hooper et al., 2017) and MuLocDeep protein localization prediction tool (Jiang et al., 2021; Jiang et al., 2023). The mitochondrial localization was previously validated, or predicted, for the majority of proteins uniquely detected in the mitochondrial fraction, with plastidic proteins being the main contaminants, similar to observations from other studies employing the mitochondrial isolation protocol (Rao et al., 2017). HS induced extensive changes in composition of both cytosolic and mitochondrial fractions, and functional enrichment analysis underlined proteasome and protein folding as key mediators of stress and also, potentially, the PCD response. Unfolded protein response is generally a pro-survival mechanism but may also induce PCD in plants subjected to severe or chronic stress levels (Williams et al., 2014; Liu and Howell, 2016). Likewise, although the disruption of proteasome function may lead to PCD activation (Kim et al., 2003), multiple studies report pro-PCD proteasome roles at different stages of plant PCD (Vacca et al., 2007; Pajerowska-Mukhtar and Dong, 2009). Protein homeostasis machinery seems, therefore, to be tightly interconnected with regulation of life and death decisions in plant cells. Given that the release of specific mitochondrial proteins, such as cyt *c*, SMAC, Omi, and AIF into the cytosol activates a critical series of events during PCD in animal cells (Bock and Tait, 2020), we attempted to identify candidate proteins similarly involved in plant PCD by determining mitochondrial proteins undergoing controlled release into the cytosol upon stress insult. A total of 125 proteins with mitochondrial localization according to SUBA (Hooper et al., 2014) were translocated into cytosol in response to HS, as indicated by their decreasing and increasing abundance in the mitochondrial and in the cytosolic fractions, respectively (**Supplementary Table S4A**). For 113 of these proteins, the downregulation in the mitochondrial fraction exceeded that observed for the mitochondrial matrix structural marker IDH2 (Rikhvanov et al., 2007; Lopez-Huertas and del Río, 2014; Lee et al., 2019), suggesting that their presence in the cytosol is not simply a consequence of mitochondrial degradation in response to heat but instead a more selective release from the mitochondria into the cytosol. As anticipated, this included not only cyt *c*, whose release is well documented during plant PCD (Balk et al., 1999; Balk and Leaver, 2001; Virolainen et al., 2002; Yao et al., 2004; Morimoto et al., 2007), but also other proteins that could play a role in PCD regulation in plants such as NDPK3 and HX-1. The nuclease activity inhibited by ATP and specific to structured supercoiled nucleic acids was previously demonstrated for the NDPK3 ortholog from pea mitochondria (Hammargren et al., 2007) with the authors hypothesizing that NDPK3 located in soluble IMS could be activated by a decreasing ATP concentration during plant PCD, released from mitochondria and translocated into the nucleus for DNA degradation (Hammargren et al., 2007). Although our dataset supports the early PCD-associated release of NDPK3 from plant mitochondria, further investigation is required to determine its role in PCD regulation. HX-1 has previously been mainly studied as a glucose sensor in plants (Moore et al., 2003) but has been also reported to play a role in PCD regulation (Kim et al., 2006; Godbole et al., 2013). Although further studies are required, the observed translocation of HX-1 into the cytosolic fraction could be an event amplifying the PCD signal, similar to animal models where dissociation of HX-1 from the OMM was demonstrated to induce VDAC1-dependent mitochondrial permeability transition pore opening and consequently apoptotic cell death (Abu-Hamad et al., 2008).

Potential similarities between animal and plant PCD programs were further highlighted by comparing the list of proteins translocated from the mitochondria into the cytosol in the present study to those released from mouse mitochondria following chemically induced permeability transition (Patterson et al., 2000). This comparison suggested a potential role in plant PCD regulation for proteins that were already identified as cell death modulators in animal models, such as adenylate kinases (Köhler et al., 1999; Lee et al., 2007). Similarly, the release of a fraction of the mitochondrial antioxidant pool, including enzymes such as manganese superoxide dismutase 1 (MSD1), thioredoxins, peroxiredoxins, and ferredoxins, could indicate a relative deprotection of mitochondrial membranes from oxidative reactions, as previously suggested in the case of animal cell death (Patterson et al., 2000) and, therefore, result in a stronger PCD induction signal. The release of mitochondrial HSPs (HSP10, HSP60, and mtHSP70) was also observed. These HSPs are key mitochondrial chaperones with essential functions in mitochondrial biogenesis, such as import and folding of proteins (Böttinger et al., 2015), and, hence, their release may lead to accumulation of mitochondrial damage and loss of mitochondrial function. This is supported by a previous study reporting increased cell viability following heat and H_2_O_2_ treatment resulting from overexpression of mtHSP70 in rice (Qi et al., 2011). Data presented herein are also in line with a previous study using *Arabidopsis* cell suspension culture that, although not focused on the early stages of PCD induction, nevertheless, demonstrated that the lethal levels of heat cause upregulation of mitochondrial HSP60 synthesis and induce its release from mitochondria during 2 h of stress recovery (Rikhvanov et al., 2007). The immunoblotting performed here suggests that this controlled release of HSP60 occurs early during PCD induction but not in response to sub-lethal levels of HS. In addition to causing mitochondrial damage and, this way, amplifying the PCD-inducing signal, the released HSP60 could also play a PCD regulatory role in the extramitochondrial locations. This is plausible considering data from animal model systems where the early translocation of HSP60 into cytosol was recorded during apoptosis induced in mammalian cells (Samali et al., 1999; Chandra et al., 2007). Released HSP60 seemed to promote cell death by accelerating caspase-3 activation in the cytosol (Samali et al., 1999; Chandra et al., 2007). In contrast, *de novo* accumulation of cytosolic HSP60 appears to play a pro-survival role (Chandra et al., 2007; Chun et al., 2010). Consequently, it was proposed that the pro-survival or pro-death function of HSP60 in the cytosol is dependent on its origin, with a mitochondrial release of HSP60 promoting apoptosis (Chandra et al., 2007; Huang and Yeh, 2019). Exploring the potential analogous, localization-dependent PCD-related roles of mitochondrial HSP60 is, therefore, recommended to further elucidate the cell death signaling in plants. In conclusion, although the functional involvement in plant PCD regulation remains elusive for the array of identified proteins released from mitochondria, the generated dataset will provide a starting point for the future research efforts in this area.

### 4.2 Changes to cytosolic proteome upon PCD induction

The present study identified upregulation of many proteins of non-mitochondrial origin in the cytosolic fraction (**Supplementary Table S4B**), enriching GO terms related to both pro-PCD and pro-survival signaling. For example, not only the positive PCD regulator metacaspase 1 (AT1G02170) but also its inhibitor SERPIN1 (Lema Asqui et al., 2018) were among the upregulated cytosolic proteins. Upregulation of ACD2 (accelerated cell death 2) protein in the cytosolic fraction following PCD-inducing heat treatment was also observed, in line with a previously reported shift in ACD2 localization during pathogen-induced cell death, from being largely in chloroplasts to partitioning to chloroplasts, mitochondria, and cytosol (Yao and Greenberg, 2006). Another upregulated cytosolic protein, BAG4, was previously suggested to inhibit plant PCD and to function in stress tolerance in plants (Doukhanina et al., 2006; Kabbage et al., 2017; Thanthrige et al., 2020). Spermidine synthases 1, 2, and 3 (AT1G23820, AT1G70310, and AT5G53120); key enzymes from the polyamine synthesis pathway (Liu et al., 2015); and proteinase inhibitor cystatin B (AT3G12490, Wong et al., 2006) were among upregulated cytosolic proteins previously implicated in regulation of stress tolerance in plants. Intriguingly, the data also suggest cytosolic uptake of extracellular proteins, with many of them exhibiting hydrolase activities, such as subtilisin-like proteases and one ribonuclease RNS1. Their translocation from the apoplast into cytosol may be a critical step in execution of plant PCD, as previously described for phytaspase, a proteolytic enzyme that is secreted from healthy plant cells but upon induction of PCD reimported from the intercellular space into the dying cells for the degradation of intracellular proteins (Chichkova et al., 2010). Similarly, the extracellular ribonuclease S-RNAse has been previously shown to promote PCD associated with pollen incompatibility after internalization *via* a yet unconfirmed mechanism (Williams et al., 2015). Indeed, overexpression of one of the proteases identified here, SBT2.2, was reported to induce cell death dependent on the plasma membrane protein ACD6 (Zhu et al., 2021), and RNS1 was previously identified as a positive regulator of fumonisin B1-induced PCD (Goodman et al., 2022). Re-importing hydrolytic enzymes back to the cytosol to drive the cell content degradation may, therefore, be a common strategy during plant PCD. Another potential explanation for the observed increase in abundance of extracellular proteins in the cytosol upon PCD induction is rapid inhibition of their secretion in response to stress stimulus. Future research is required to further investigate both possibilities.

We also observed a downregulation of >1,300 proteins in the cytosolic fraction following a PCD-inducing heat treatment (**Supplementary Table S6A**), enriching GO terms related to stress response, proteasome structure and function, HSPs, and DNA repair and translation. We attribute this large-scale downregulation of proteins from the cytosolic fraction not only to the possible proteolytic degradation of cell contents associated with PCD driven by the observed increase in proteins with hydrolytic activity but also to sequestration of proteins into SGs and other protein aggregates that are removed from cytosolic fraction and may copurify with the mitochondria during the isolation protocol. The presence of SG marker proteins and the comparisons to SG proteome (Kosmacz et al., 2019) support this hypothesis and highlight that the role of SGs and other cytoplasmic protein aggregates should be further explored in context of balancing life and death decisions of plant cells and carefully considered by future studies employing subcellular fractionation protocols in combination with stress treatments.

The immunoblotting experiments confirmed downregulation of cytosolic HSP70 after the HS and suggested that it at least partially copurifies with the mitochondrial fraction after PCD-inducing treatment, which could be explained by interactions between this molecular chaperone and misfolded protein aggregates (Żwirowski et al., 2017). Lower concentrations of cytosolic HSP70 could also promote PCD signaling in plants. In animals, HSP70 counteracts the death signaling by inhibition of caspase-3-like proteases (Jäättelä et al., 1998). In tomato and tobacco, HSP70 accumulation stimulated by mild heat minimizes cell death rates after subsequent SA exposure (Cronjé and Bornman, 1999; Cronjé et al., 2004). HSP70 is also involved in developmental PCD at perforation sites of lace plant leaves (Rowarth et al., 2020).

### 4.3 Effect of lanthanum chloride on HS-induced changes to mitochondrial and cytosolic proteomes

In line with previously published data (McCabe et al., 1997; Kacprzyk et al., 2017), the LaCl_3_ applied before HS resulted in almost complete inhibition of PCD, with cells exhibiting delayed viability loss and dying *via* necrosis instead of PCD. Globally, the effect of LaCl_3_ on protein composition of isolated fractions appeared limited, and we did not detect any effect of LaCl_3_ on the release of mitochondrial proteins triggered by heat. We hypothesize that LaCl_3_ acts downstream of the mitochondrial cell death initiation signal in our system, as indeed there were certain differences in changes of protein abundance induced by HS in cells treated in the presence of LaCl_3_. The upregulation of 31% proteins in the cytosolic fraction was inhibited in the presence of LaCl_3_. Interestingly, this group included several proteins from the WD40/transducin family that is known to mediate cell death and survival in animal models (Zhang and Zhang, 2015), and GO terms for this group of proteins were enriched for hydrolase and peptidase activities. LaCl_3_ also partially inhibited upregulation of nuclear, plastidic, peroxisomal, and vacuolar proteins in the cytosolic fraction following the HS, suggesting that the cells heat-treated in presence of LaCl_3_ better preserve the integrity of these organelles. The import of nine (out of 33) extracellular proteins, including SBT2.2 protease, into the cytosol, was also inhibited in the presence of LaCl_3_. Overall, this pattern may indicate that LaCl_3_ likely delayed/inhibited the execution/degradation phase of plant PCD. The downregulation of proteins in the cytosolic fraction was also partially inhibited when HS was performed in the presence of LaCl_3_, with several of them being previously linked to cell death and/or stress responses in plants (e.g., BAG7, LBA1, and LYSOPL2) (Gao et al., 2010; Williams et al., 2010; Raxwal et al., 2020). Moreover, functional enrichment analysis of proteins not downregulated by HS in the presence of LaCl_3_ highlighted the terms related to gene expression and translation, indicating that LaCl_3_-treated cells may maintain the ability to activate pro-survival responses dependent on *de novo* gene expression and protein synthesis. Notably, BAG7 was the only protein showing an opposite direction of regulation in the cytosolic fraction due to LaCl_3_ treatment, downregulated by HS in the cytosolic fraction but upregulated if HS was performed in presence of LaCl_3_. Previous studies suggest that, when localized in the ER, BAG7 acts as a cell death suppressor (Williams et al., 2010), but it may also promote immunity-related cell death after proteolytic cleavage and translocation to nucleus (Zhou et al., 2021). The data presented here, therefore, warrant further detailed investigations into localization-dependent roles of this protein in cell death regulation in plants.

## 5 Conclusions

**Figure 5:**
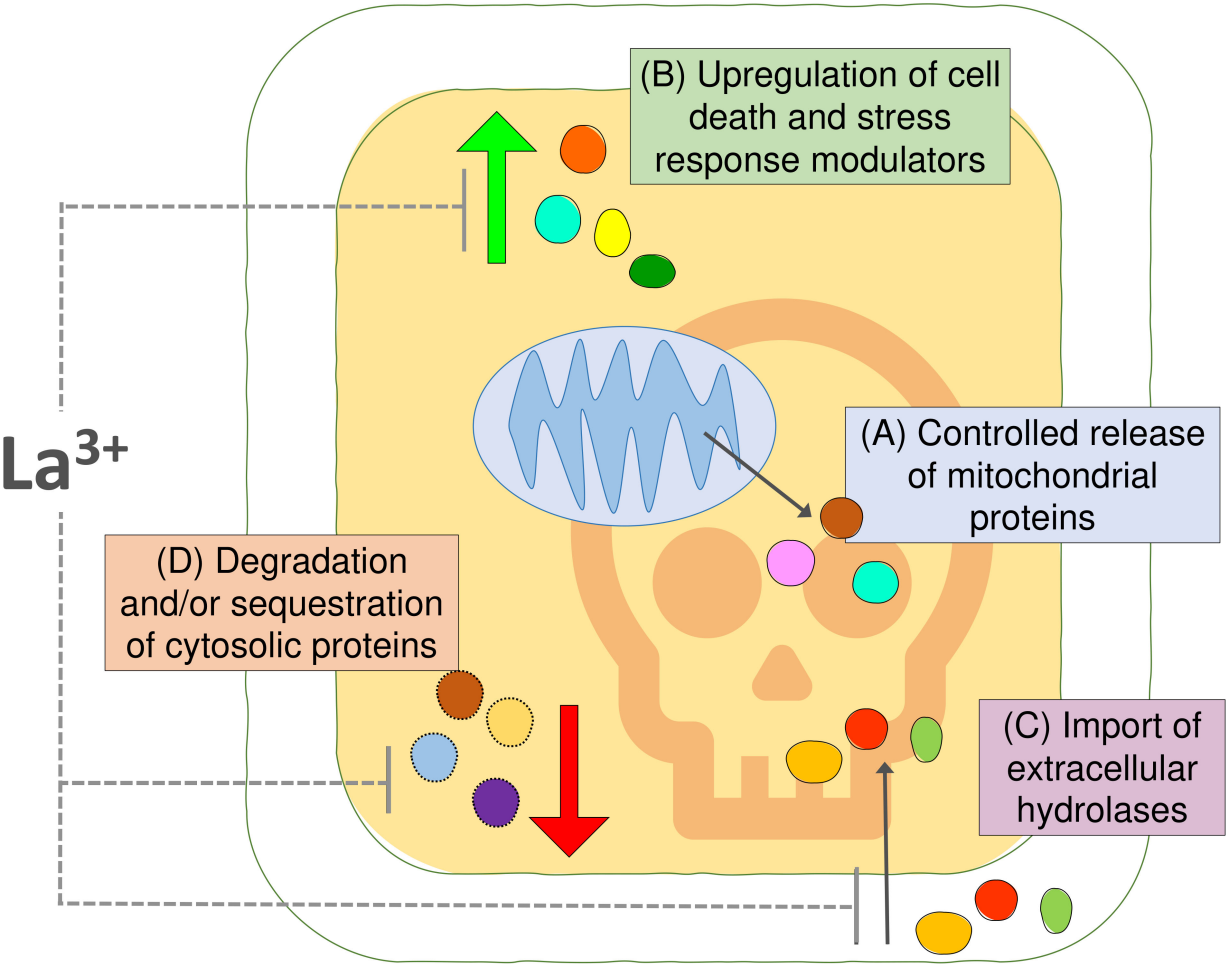
Summary figure. **(A)** Comparisons of the proteomic profiles of cytosolic and mitochondrial fraction from *Arabidopsis* suspension cells identified 113 proteins undergoing controlled release from mitochondria upon the PCD-inducing stress insult. **(B)** The changes in the cytosolic fraction revealed upregulation of proteins putatively involved in cell death and stress response and **(C)** suggested that import of extracellular hydrolases into the cytosol may promote degradation of cellular content during PCD. **(D)** Degradation and/or sequestration of proteins from the cytosolic fraction indicate another layer of regulation of plant PCD process. The calcium channel blocker and PCD inhibitor LaCl_3_ had no effect on the release of proteins from mitochondria in this system; however, it had partially prevented changes observed in the cytosolic fraction after PCD induction.

We used an *Arabidopsis* cell suspension model and MS proteomics to characterize changes in protein abundance and localization associated with the early stages of HS-induced PCD (**Figure 5**). We identified 113 proteins that may undergo controlled release from mitochondria following the PCD-inducing HS, including the IMS-localized NDPK3 with nuclease activity and *Arabidopsis* homologs of mitochondrial proteins with previously identified roles in regulation of animal cell death, such as adenylate kinases and HSP60. Future studies are required to differentiate proteins with a functional cytosolic role in plant PCD process from the innocent bystanders released from plant mitochondria. Furthermore, we characterized the proteome changes in the cytosolic fraction resulting from PCD-inducing stress, underscoring the importance of proteasome function, chaperone-mediated protein folding, and HSPs in life-death decision in plant cells. Our data uncovered increases in cytosolic abundance of both pro-PCD and pro-survival proteins and indicated that extracellular proteins with hydrolytic activities may be reimported back to the cytosol for degradation of cell content, in a manner similar to the previously described PCD-promoting protease, phytaspase (Chichkova et al., 2010). Moreover, we hypothesize that HS-induced downregulation of proteins in the cytosolic fraction could be linked to both proteolytic degradation and sequestration of proteins into cytosolic aggregates such as SGs and that both processes may modulate the balance between pro-survival and pro-death signaling pathways during the cellular stress response. Experiments with the calcium channel blocker and PCD inhibitor LaCl_3_ suggested that, in our system, it acted downstream of the mitochondrial cell death initiation signal, by partially inhibiting stress-induced changes in protein abundance in the cytosolic fraction that may be related to PCD processes, such as upregulation of proteins with hydrolytic activity or downregulation of general translation machinery. In particular, the presence of LaCl_3_ reversed the effect of HS on ER-associated BAG7 protein that was previously suggested to have localization-dependent roles in plant PCD regulation. Collectively, we point out that the results presented here will form a resource to enable the further elucidation of the regulation of the fundamental programmed death process in plants.

## Data availability statement

The generated MS datasets and MaxQuant search output files are openly accesible via PRIDE repository under identifier PXD040584.

## Author contributions

The study idea was originally conceived by JK and PM and subsequently developed by all co-authors. The experiments were performed by JS. JC has overseen mass-spectrometry profiling and data analysis. GS advised on the western blotting experiments. The initial version of manuscript was drafted by JS and JK, and all authors (JS, JC, GS, PM, JK) contributed to the final version of this paper. All authors contributed to the article and approved the submitted version.

## Supplementary material

Supplementary Figure 1 Full images of immunoblot membranes for each antibody with respective exposure time.
